# Alcohol craving under stress in healthy young men: A randomized laboratory study

**DOI:** 10.1111/acer.70257

**Published:** 2026-02-19

**Authors:** Charlotte Wittgens, Anja Kräplin, Markus Muehlhan, Fée Ona Fuchs, Sebastian Trautmann

**Affiliations:** ^1^ Department of Psychology, Faculty of Human Science Medical School Hamburg Hamburg Germany; ^2^ ICPP Institute for Clinical Psychology and Psychotherapy Medical School Hamburg Hamburg Germany; ^3^ Section of Systems Neuroscience, Department of Psychiatry and Psychotherapy Technische University Dresden Dresden Germany; ^4^ Work Group Addictive Behaviors, Risk Analysis and Risk Management, Faculty of Psychology Technische University Dresden Dresden Germany; ^5^ ICAN Institute for Cognitive and Affective Neuroscience Medical School Hamburg Hamburg Germany

**Keywords:** alcohol craving, alcohol use disorders, experimental stress, trier social stress test

## Abstract

**Background:**

Harmful alcohol use and alcohol use disorders (AUD) are major public health issues. Alcohol craving plays a central role in AUD by increasing the likelihood of consumption. While stress is a known trigger for craving, most evidence comes from observational or clinical studies. This study aimed to investigate the causal effect of experimentally induced stress on alcohol craving.

**Methods:**

Two hundred and eighty‐eight healthy young men were randomized to either the Trier Social Stress Test or a control condition. Alcohol craving was assessed before and immediately after the task. Stress reactivity was measured via mood scales and biological (salivary cortisol and alpha‐amylase) markers. Latent mixture modeling explored variability in the effect of stress exposure on craving, and latent group differences were examined in relation to habitual alcohol use, self‐control, anxiety, depression, and emotion dysregulation.

**Results:**

Participants exposed to the stressor reported higher poststress craving compared with controls (*b* = 2.35, 95% CI [0.54, 4.16], *p* = 0.011). Within the stress group, greater mood changes (*b* = −0.53, 95% CI [−0.82, −0.24], *p* < 0.001) and biological (alpha‐amylase) stress reactivity (*b* = 0.01, 95% CI [−0.02, 0.04], *p* = 0.391) were associated with increased craving. Two latent classes emerged: one (51.4% class membership) showed a positive effect of stress on craving (stress‐related craving increase class [SCI]) (*b* = 5.37, *p* = 0.002), while the other (49.6%) showed a negative effect (stress‐related craving decrease class [SCD]) (*b* = −2.70, *p* = 0.012). The SCI class was characterized by higher anxiety and depressive symptoms, greater emotion dysregulation, and lower self‐control compared with the SCD class.

**Conclusions:**

Experimental stress can increase alcohol craving, particularly in individuals with certain psychological profiles. Craving was linked to subjective and biological stress reactivity. These findings highlight the importance of individual differences in stress responses and provide targets for more personalized studies on stress‐related craving and alcohol use.

## Graphical abstract

This study shows that acute social stress can increase alcohol craving in young men. Individuals with stronger mood and biological reactivity showed greater increases in craving. Two latent classes of stress‐craving effects emerged in exploratory analyses: one class showed stress‐induced craving increase while the other showed a decrease compared with the nonstressed control group. The classes differed in anxiety, depression, and emotion dysregulation. These findings emphasize the importance of individual differences in alcohol craving responses to acute stress.

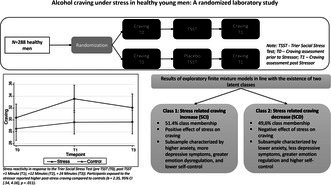

## INTRODUCTION

Harmful use of alcohol and alcohol use disorders (AUD) are among the leading public health threats, associated with immense societal and individual burden (Rehm et al., 2017). Excessive alcohol use increases the risk of various somatic conditions, including communicable and noncommunicable, digestive, and cardiovascular diseases (World Health Organization, 2018). Globally, 237 million men and 46 million women meet the criteria for AUD (World Health Organization, 2018). Although previous data indicate that gender differences in alcohol consumption are narrowing, with alcohol use among women rising sharply (Peltier et al., 2019), men continue to show higher overall rates of problematic drinking (8.6% vs. 1.7%) particularly in the 18–40 age group and are more likely to cope with stress through alcohol use, displaying stronger associations between daily stress and drinking behavior (Ayer et al., 2011; World Health Organization, 2018).

A broad range of psychological and pharmacological evidence‐based interventions for harmful alcohol use and AUD (Morley et al., 2021) support abstinence or reduced consumption. However, a substantial treatment gap persists, as many seek help only years after onset, often after severe consequences have occurred (Chapman et al., 2015). Thus, there is a need for preventive strategies targeting at‐risk populations for excessive forms of alcohol use, ideally before the onset and manifestation of harmful use patterns and AUD. This requires understanding of the underlying mechanisms leading to excessive alcohol use.

Multiple factors influence the development of excessive alcohol consumption and AUD. Stress, defined as uncontrollable or unpredictable events that exceed an organism's regulatory capacity and threaten physical or psychosocial integrity, is among the most well‐described major risk factors for excessive alcohol use and AUD (Lijffijt et al., 2014). Cross‐sectional studies indicate that individuals with AUD experience more stress than healthy controls (Coffey et al., 2016; McGrath et al., 2016). Longitudinal studies identified major life stressors, such as exposure to disasters, childhood maltreatment, and interpersonal stressors, as significant risk factors for the early onset of alcohol consumption and AUD (Cerdá et al., 2011; Keyes et al., 2011). Stress may influence alcohol use through psychological mechanisms such as self‐medication (Khantzian, 1997) and biological changes involving altered hypothalamic–pituitary–adrenal (HPA) axis and sympathetic nervous system (SNS) reactivity (Trautmann, Muehlhan, et al., 2018b). Importantly, stress plays a pivotal role not only in the development of harmful use and AUD but also in early phases of alcohol consumption, from the initiation to regular consumption (Lijffijt et al., 2014). This makes it a valuable target for preventive interventions.

Another crucial construct for understanding AUD is craving. Craving is a salient and persistent subjective experience commonly reported by individuals with harmful substance use who are trying to abstain from using substances. Craving is a hallmark of addiction, making it challenging to overcome alcohol‐seeking behavior (Sinha, 2013). Alcohol craving is frequently used as a proxy for alcohol use behaviors, being strongly associated with increased consumption and relapse risk and serves as a subjective indicator of AUD (de Bruijn et al., 2004). This association highlights the importance of addressing craving in both understanding and treating AUD. Stress plays a decisive role in this association and is often investigated as a factor for consumption and relapse (Seo & Sinha, 2014). Studies on craving within clinical samples with AUD are consistently reporting higher craving for alcohol following stress (Blaine & Sinha, 2017; Guinle & Sinha, 2020). Similarly, in heavy social drinkers (defined as regular consumers averaging one to three drinks/day for women and two to four for men without AUD), stress has been associated with increased craving and alcohol intake throughout the day (Mayhugh et al., 2018). Prospective studies found that stress significantly heightens craving, again leading to greater alcohol consumption the following day (Wemm et al., 2019), and is a key trigger for alcohol craving in cue exposure therapy (Ghiţă et al., 2019). These real‐world findings highlight the strong link between stress and alcohol craving. To investigate causal effects, experimental laboratory studies with controlled condition to manipulate stress and minimize confounding variables are needed. Stressful environments can trigger alcohol craving, partly through elevated cortisol levels, which enhance alcohol's reinforcing properties and intensify the urge to consume (Seo & Sinha, 2014). Thus, alcohol craving may mediate the relationship between stress exposure and alcohol use. However, most experimental research on the effects of laboratory‐induced stress on craving focused on clinical populations (McGrath et al., 2016; Thomas et al., 2011), limiting their relevance for prevention. Available studies in nonclinical populations have similarly demonstrated that acute stress can increase alcohol craving and, in some cases, alcohol consumption (Blaine et al., 2019), involving different psychological mechanisms, such as alcohol cue reactivity (Blaine et al., 2019), risk‐taking (Clay et al., 2018), and decision‐making (Clay & Parker, 2018). These studies have made important contributions to understanding stress‐related drinking behavior; however, their relatively modest sample sizes highlight the need for further large‐scale investigations to confirm and extend these findings. Moreover, potential interindividual variability in the impact of stress on craving—despite strong theoretical and empirical evidence supporting heterogeneous stress responses—has not yet been systematically examined.

This study investigates the effects of stress exposure on alcohol craving in healthy men in a randomized controlled laboratory study. We hypothesized (1) that stress exposure would increase alcohol craving compared with a control condition, and (2) that stress‐induced craving would be positively associated with both subjective and biological stress reactivity. As an exploratory question that was added post hoc after data collection, we investigated potential heterogeneity in the association between stress exposure and alcohol craving by exploring evidence for distinguishable latent classes, and sought to characterize them regarding psychological and alcohol‐related variables.

## MATERIALS AND METHODS

### Study design

A comprehensive study protocol describing the study design, procedures, and planned analyses was published during the recruitment phase, ensuring transparency of the methodological approach (Wittgens et al., 2022). In brief, the current study is a randomized controlled laboratory study that was conducted in the Department of Psychology at Medical School Hamburg between December 2018 and August 2022. The study consists of two parts, an online screening for eligibility check and a main study in the laboratory. Participants were randomly assigned to either an experimental (acute stress exposition) or a control condition (no stress) with subsequent measures of stress reactivity and the assessment of psychological and alcohol‐related variables.

### Participants

Two hundred eighty‐eight healthy men participated in the study. The present study focused exclusively on male participants to reduce potential variability associated with well‐documented sex differences in stress physiology and affective responding. Prior research indicates that women typically exhibit greater HPA axis sensitivity, heightened cortisol reactivity, and stronger subjective emotional responses to stress compared with men (Guinle & Sinha, 2020). These biological and psychological differences in stress reactivity and coping strategies could introduce additional variability and confound interpretation of sex‐specific patterns in stress‐related drinking behavior. A primary objective was to ensure a sufficiently powered sample size, which has often been lacking in previous comparable studies, thereby limiting the replicability of findings from randomized experiments (Peters, 2017). However, achieving equally large samples for both males and females was not feasible within the available resources; therefore, the study focused on male participants. Limitations of this approach considering recent developments in gender differences in substance use are discussed later on. The age range was between 18 and 40 years mean age *M* = 24.92 (SD = 4.4). Individuals older than 40 years were not included to reduce variance in reaction times and biological indicators (Kudielka et al., 2004). Participants were recruited via e‐mail advertisements, personal contacts and flyers in university and public settings. Initial eligibility was ascertained through an online screening prior to an invitation for the main assessment in the laboratory. The online screening included questions to basic demographic variables, as well as all inclusion and exclusion criteria of the study. The inclusion criteria were male sex, age between 18 and 40 years, and at least occasionally (in the following referred to as habitual alcohol consumption) drinking alcohol with beer as their favorite drink (Wittgens et al., 2022). Additionally, a hair length of at least 2 cm was required for cumulative cortisol secretion analysis, which is not part of this article. Individuals were excluded when they were screened positive for lifetime psychotic symptoms, substance use disorder or current psychological or psycho‐pharmacological interventions. Additionally, individuals with somatic diseases (e.g., cardiovascular disease, asthmatic disease, and hypertension) that might confound the variables of interest were excluded from participation.

### Average habitual alcohol consumption

For assessment of average habitual alcohol consumption, all participants filled out a retrospective report of average daily alcohol intake for the past 30 days (web‐based Self‐Administered Timeline Follow Back, STLFB) prior to the laboratory assessment (Collins et al., 2008). The STLFB assesses typical daily alcohol intake as well as situational variations and provides information on regular and risky drinking habits (Collins et al., 2008). The average daily alcohol consumption in grams was calculated. The STLFB records alcohol consumption separately for weekdays and weekends, and special drinking occasions, when participants indicate drinking more than typical. The online STLFB shows high correlations with the interview format in measures of alcohol use (Hareskov Jensen et al., 2023). Compared with widely used quantity/frequency measures, the STLFB is superior in capturing situational variations and binge drinking episodes (Collins et al., 2008).

### Stress induction

Participants were exposed to the Trier Social Stress Test (TSST) (Kirschbaum et al., 1993) or a control condition without stress exposure. The TSST is one of the most commonly employed paradigms for stress induction and provides a reliable and ecologically valid stressor (Kirschbaum et al., 1993). It involves elements of social evaluative threat and uncontrollability. The test consists of three 5‐min parts: a preparation period, a free speech for a job interview, and an arithmetic task, all performed in front of a two‐person audience. The TSST results in significant changes in the HPA axis and the autonomic stress response (Dickerson & Kemeny, 2004). Participants in the control condition took part in a placebo TSST. The placebo TSST is also divided into equal parts of 5 min for preparation, a speech about the last holiday, and an easy arithmetic task but without stress exposure for the participants (Het et al., 2009). The main difference to the TSST is participants being alone with no audience while performing the tasks. Hence, there are no components of evaluative threat and uncontrollability.

### Craving

The alcohol craving questionnaire revised (ACQ‐r) was used to assess craving (Raabe et al., 2005). The ACQ‐r is a 12‐item self‐report instrument using a 7‐point Likert response format from 1 (strongly disagree) to 7 (strongly agree) and focuses on the duration, frequency, and intensity of alcohol craving, with higher scores indicating stronger craving. Craving was assessed before (T0), directly after the stressor (T1), and 24 min later (T3), of which the first two measurements are relevant for the investigation of the average causal effect of stress on craving. The internal consistency in the present sample was α = 0.93.

### Stress reactivity

**FIGURE 1:**
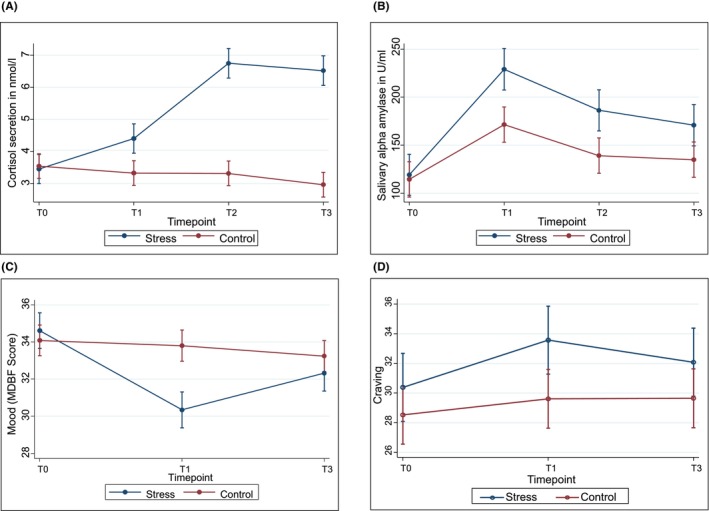
Stress reactivity in response to the Trier Social Stress Test (pre‐TSST (T0), post‐TSST +1 min (T1), +12 min (T2), + 24 min (T3)). (A) cortisol; (B) alpha‐amylase; (C) mood; (D) craving.

Self‐reported and biological stress reactivity was measured. First, self‐reported mood was assessed using the Multidimensional Mood State Questionnaire (MDBF) (Steyer et al., 2004). The MDBF Mood scale consists of 8 items and uses a 5‐point Likert response format with lower scores indicating poorer mood condition. Self‐reported mood was assessed at three timepoints throughout the main assessment to investigate mood changes over time (Figure 1). Furthermore, stress‐induced cortisol secretion and alpha‐amylase as a measure of SNS activity were collected using four saliva samples throughout the main assessment as biological indicators of stress reactivity (Wittgens et al., 2022). An increase in cortisol and alpha‐amylase indicated an increase in biological stress reactivity. For this purpose, Salivettes “blue cap” (Sarstedt, Nümbrecht Germany) with synthetic swabs were used. Participants had to provide saliva samples directly before and at the three timepoints after stress exposure or control condition. Saliva samples were stored at −20°C in a laboratory freezer. After thawing, saliva samples were centrifuged for 10 min at 4000 rpm. Salivary cortisol concentrations were determined using a commercially available chemiluminescence assay (CLIA, IBL‐Hamburg, Germany) (Wittgens et al., 2022). Salivary alpha‐amylase activity was detected by using an in‐house enzyme kinetic method according to the protocol described in Rohleder and Nater (2009).

### Psychological characteristics

Participants completed a comprehensive baseline assessment (questionnaire package) (Wittgens et al., 2022). Anxiety was assessed using the State‐Trait Anxiety Inventory (STAI‐T) (Grimm, 2009). The STAI‐T consist of 20 items using a 4 point Likert scale measuring general, long‐term anxiety proneness. Higher scores indicate greater anxiety. Depression was assessed using the Becks Depression Inventory (BDI) (Beck et al., 2000). The BDI consists of 21 items and is rated on a 4‐point scale, with higher scores indicating greater symptom severity. Furthermore, the Brief Self‐Control Scale (BSCS) (Tangney et al., 2004) that measures general trait self‐control—the ability to regulate impulses, resist temptations, and maintain goal‐directed behavior was applied. It consists of 13 items and is rated on a 5‐point Likert scale with higher scores indicating greater self‐control. Drinking motives were examined using the Drinking Motive Questionnaire‐Revised (DMQ‐R) (Kuntsche & Kuntsche, 2009) with 20 items on a 5‐point Likert scale which differentiate between 4 subtypes of drinking motives. Lastly emotion regulation was assessed using the Difficulties in Emotion Regulation Scale (DERS) (Gratz & Roemer, 2003). The DERS includes 36 items and differentiates between six domains of emotion regulation. Items are rated on a 5‐point Likert scale with higher scores indicating greater difficulties in emotion regulation.

### Procedure

All procedures of the main study have been described in detail elsewhere (Wittgens et al., 2022). The study was conducted in accordance with the revised declaration of Helsinki and Good Clinical Practice Guidelines and approved by the Institutional Review Boards of Technische Universität Dresden (EK 522122016) and Medical School Hamburg (MSH‐2020/114). All participants provided written informed consent. Procedures relevant for the current investigation are as follows. The main assessments in the laboratory were conducted between 2 p.m. and 8 p.m. for reduction of variance in biological measures (saliva cortisol and alpha‐amylase) due to diurnal rhythms (Miller et al., 2016). Absence from alcohol was tested with a breathalyzer “ACE one” (*ACE instruments*, Freilassing, Bayern, Germany). If the result showed any value above zero, participants were immediately excluded from the study. First, participants were informed about the study procedures and that they were able to taste alcoholic drinks (beer) after the experimental procedure with the aim to assess the effects of stress and personal variables on taste (cover story). Then, participants filled out baseline questionnaires followed by the Trier Social Stress Test (TSST) or placebo TSST. A deviation from the standard TSST protocol in the present study was the fact that the stress condition was continued for additional 30 min after completion of the TSST during several behavioral tasks, which are not part of the current paper. During these tasks, participants were instructed that the panel will keep observing their performance to prevent a decline in stress levels. Four saliva samples were taken during the assessment prestress exposition (T0), and at three times poststress exposition +1 min (T1), +12 min (T2), +24 min (T3) to examine participants changes in cortisol secretion and alpha‐amylase activity over time. Self‐reported craving was measured directly before (T0) and after the stressor (T1). Mood was measured directly before the stressor (T0), as well as at two further time points afterward (T1 and T3). Since cortisol rises immediately after stress exposure and peaks at 15–20 min (Russell & Lightman, 2019) timepoint (T2) was used exclusively for measuring cortisol and additionally alpha‐amylase in this regard. At the end of the laboratory session, participants were debriefed about the true purpose of the study and received a compensation of 20€.

### Statistical analyses

A series of simulation‐based a priori power analysis was conducted assuming analysis groups of *n* = 200, which resulted in sufficient statistical power (>0.80) (see Wittgens et al., 2022). A treatment check was conducted to investigate whether the TSST evoked a stress reaction as intended in terms of changes in cortisol and alpha‐amylase secretion as well as changes in self‐reported mood compared with the control group. Mixed effects regression with random intercept parameter and fixed effects of time (measurement timepoints) were conducted for this purpose to account for regression to the mean and missing values. To analyze test the main hypothesis of the effect of stress exposure on craving, we modeled linear regressions with predicting poststressor craving with group membership (stress vs. control), adjusting for baseline craving. Although the comparison of poststress craving between the randomized groups would be sufficient to test the causal effect, we adjusted for baseline values since this provides advantages in statistical power (Holmberg & Andersen, 2022). We also tested whether this effect would be moderated by average daily alcohol consumption (Lijffijt et al., 2014). To investigate associations between stress‐induced changes in craving and stress reactivity in the stress group, we first calculated the area under the curve with respect to increase (*AUCi*) for cortisol and alpha‐amylase over the four measurement timepoints. The AUCi is a cumulative measure of reaction dynamics over time that considers the individual input values of each stress marker (Fekedulegn et al., 2007). We then ran linear regression models for the stress group, with *AUCi* as independent and poststressor craving as dependent variable, adjusting for baseline craving and average daily alcohol consumption. Similar models were run for indicators of subjective stress, adjusting for poststressor and baseline values of subjective stress.

We further conducted a series of exploratory analyses to investigate potential heterogeneity in the association between stress exposure and craving using finite mixture modeling. In finite mixture modeling, the observed data are assumed to belong to unobserved subpopulations (classes), and mixtures of regression models are used to model the outcome of interest (Deb, 2012). After fitting the model, class membership probabilities can be predicted for each observation. We ran finite mixture models with one, two, and three latent classed and compared fit indices to select the best class solution. To be able to analyze potential characteristics of individuals from different latent classes of the effect of stress exposure on craving, we allocated individuals to the class with the highest predicted class probability. Potential differences between classes regarding habitual alcohol use, self‐control, drinking motives, trait anxiety, depression symptoms, and emotion regulation were analyzed with linear regressions. All statistical analyses were performed using Stata 15.1.

## RESULTS

### Sample characteristics

Mean age of the sample was *M* = 24.98 years (SD = 4.5). Participants were mostly high‐school and university students. Table 1 shows the baseline sample characteristics by experimental group. There were no significant differences found between the stress and control groups.

**TABLE 1:** Sample characteristics captions.

*M* (SD)	Stress group	*N*	Control group	*N*
Age	24.53 (4.53)	123	25.32 (4.53)	165
Educational degree (%)		121		164
University	32.23		40.24	
High‐school	57.85		50.00	
Junior high‐school	4.13		6.71	
Middle school	3.31		1.22	
Other				
**Stress indicators (T0)**				
Mood (MDBF)	34.61 (4.61)	123	34.09 (4.97)	165
Cortisol, nmol/L (T0)	3.45 (2.13)	116	3.52 (2.49)	159
Amylase, U/mL (T0)	117.03 (84.43)	116	107.97 (77.47)	154
Cortisol AUCi	66.38 (64.55)	100	−6.54 (44.19)	152
Alpha‐amylase AUCi	2487.31 (2359.80)	100	1145.70 (2129.52)	146
Craving (ACQ‐r) (T0)	30.38 (13.05)	123	28.53 (11.72)	165
Average daily alcohol consumption g (STLFB)	19.91 (17.16)	115	20.20 (20.24)	161

Abbreviations: ACQ‐r, alcohol craving questionnaire revised; AUCi, area under the curve with respect to increase; MDBF, Multidimensional Mood State Questionnaire (score range: 8–40); nmol/L, nano mol per liter; STLFB, Self‐Administered Timeline Follow Back; U/mL, unit per milliliter.

### Treatment check

The TSST elicited a stress response in both self‐reported and biological measures. The time × group interaction showed a higher cortisol secretion in the stress group between the reference time point before the TSST (T0) and all three poststress measurement timepoints (T1) (*b* = 1.17, 95% CI [0.65; 1.68], *p* < 0.001) (T2) (*b* = 3.52, 95% CI [3.01; 4.04], *p* < 0.001) and fourth (T3) (*b* = 3.64, 95% CI [3.12; 4.16], *p* < 0.001) time point, compared with the control group (Figure 1A,B). The *AUCi* for cortisol was *M* = 66.38, SD = 64.55 in the stress group, which was higher than the control group (*M* = −6.54, SD = 44.19, *b* = 72.92, 95% CI [59.43; 86.41], *p* < 0.001). Furthermore, there was a higher alpha‐amylase activity in the stress group between baseline and all three poststress measurement timepoints (T1) (*b* = 52.81, 95% CI [31.87; 73.75], *p* < 0.001), (T2) (*b* = 42.39, 95% CI [21.55; 63.23], *p* < 0.001), (T3) (*b* = 31.23, 95% CI [10.27; 52.18], *p* = 0.003) compared with the control group. *AUCi* for alpha‐amylase was (*M* = 2487.31, SD = 2359.8), which was again higher than the control group (*M* = 1145.7, SD = 2129.52, *b* = 1341.62, 95% CI [772.51; 1910.72], *p* < 0.001). Participants in the stress group experienced a stronger decrease in mood between baseline (T0) and second (T2) (*b* = −3.99, 95% CI [−5.78; −2.19], *p* < 0.001) and third (T3) poststress measurement (*b* = −1.44, 95% CI [−2.41; −0.48], *p = 0*.003), compared with the control group (Figure 1C).

### Associations between psychosocial stress, stress reactivity and craving

The stress group showed significantly higher craving following stress exposure than the control group, adjusted for baseline values (*b* = 2.35, 95% CI [0.54; 4.16], *p* = 0.011). As some studies have linked alcohol consumption to changes in stress responses and motivational processes associated with craving (Lijffijt et al., 2014), we investigated whether the effect of stress exposure on alcohol craving differed as a function of average daily alcohol use. There was no evidence for such a moderating effect (*b* = 0.006, 95% CI [−0.10; 0.11], *p* = 0.897). Among participants of the stress group, there was no significant association between cortisol reactivity (*AUCi*) and craving (*b* = 0.01, 95% CI [−0.02; 0.04], *p* = 0.391). Alpha‐amylase reactivity (*AUCi*) (*b* = 0.001, 95% CI [0.0004; 0.002], *p* = 0.039) and worse poststress mood (*b* = −0.57, 95% CI [−0.88; −0.25], *p* = 0.001) were significantly associated with poststress craving.

### Exploratory analyses

**FIGURE 2:**
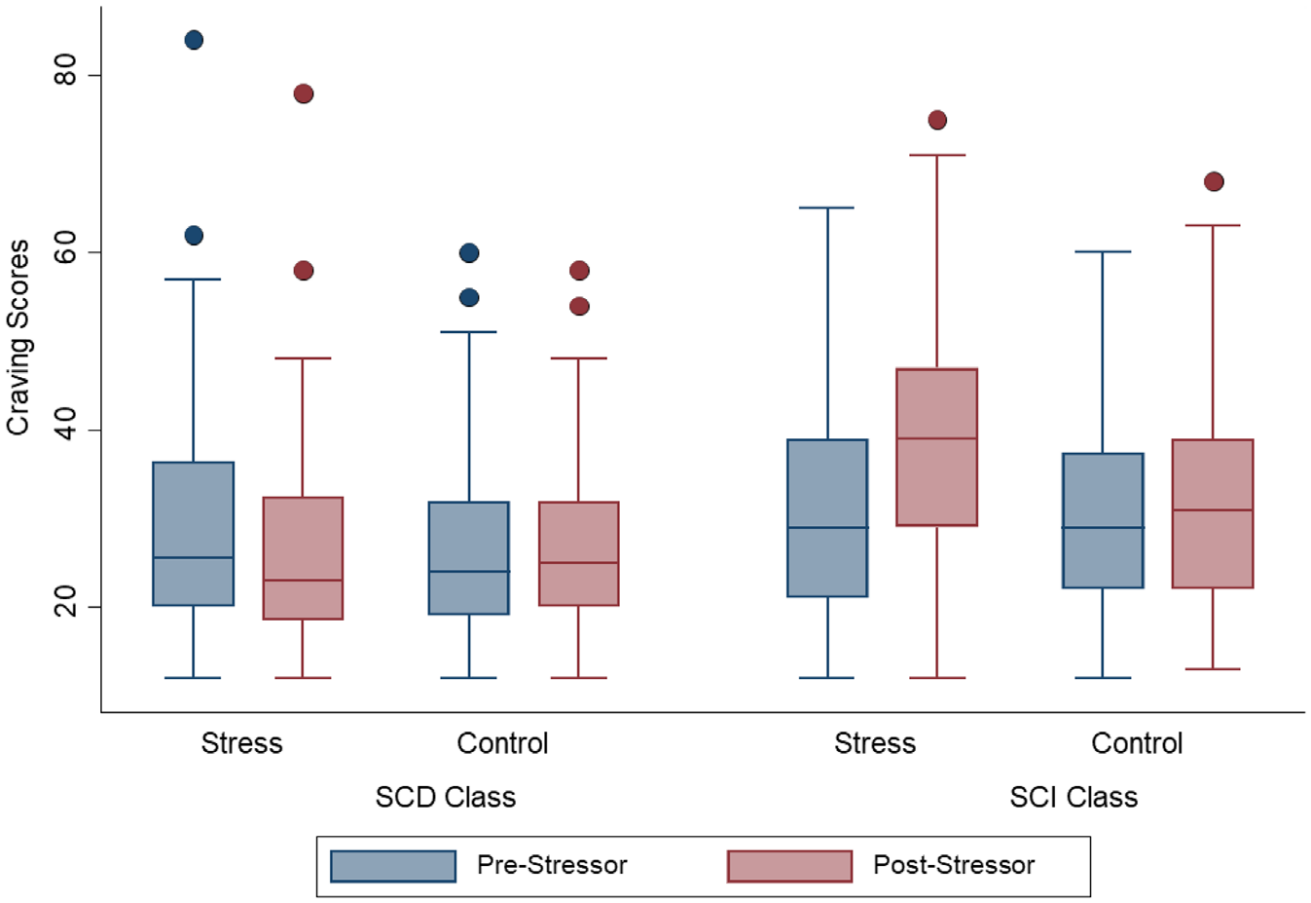
Boxplot of craving scores pre‐ and poststress by latent class (SCD vs. SCI) and experimental condition (stress vs. control group). SCD—stress‐related craving decrease class (*b* = −2.70, 95% CI [−4.81; −0.59], *p* < 0.012); SCI—stress‐related craving increase class (*b* = 5.37, 95% CI [1.99; 8.75], *p* < 0.002).

Model fit for different class solutions of finite mixture regression models analyzing unobserved heterogeneity in the effect of stress exposure on craving are shown in Table 2. Consistent with common practice, we prioritized Bayesian information criterion (BIC) over Akaike information criterion (AIC) for class enumeration (Weller et al., 2020). BIC supported a two‐class solution, whereas AIC suggested three classes. Entropy was examined as a secondary index of model fit: although the three‐class model showed a small improvement, overall separation remained modest and thus did not outweigh BIC's preference for two classes. In the three‐class model, model‐based prevalences were 16%, 42%, and 42%, and assigned (modal) class sizes were 22 (7.8%), 162 (57.5%), and 98 (34.8%). Average posterior probabilities indicated acceptable certainty for Classes 1 and 3 (diagonals 0.75 and 0.81) but lower certainty for Class 2 (0.63), with leakage toward Class 3. Balancing parsimony, fit, and classification quality, we retain the two‐class solution as primary and present the three‐class model as a sensitivity analysis for the primary effect of interest (stress exposure on alcohol craving). In the two‐class solution, the classes differed significantly in baseline craving levels, with the first class (stress‐related craving decrease class, SCD) SCD = 27.48 (SD = 11.89) and the second class (stress‐related craving increase class, SCI) SCI = 30.74 (SD = 11.89); *b* = 3.26, 95% CI [0.47; 6.05], *p* = 0.002. Following stress exposure, participants in the first class showed a decrease in craving relative to the control group (*b* = −2.70, 95% CI [−4.81; −0.59], *p* < 0.012) (therefore labeled as stress‐related craving decrease class, SCD), while participants in the second class showed an increase in craving (*b* = 5.37, 95% CI [1.99; 8.75], *p* < 0.002) (therefore labeled SCI) (Figure 2). There was no difference in habitual alcohol consumption (*b* = 0.75, 95% CI [−3.71; 5.22], *p* = 0.740) or coping drinking motive (*b* = 0.71, 95% CI [−0.01; 1.43], *p* = 0.052) between the two classes. Analyses did, however, reveal that participants in the SCI class had higher trait anxiety (*b* = 3.91, 95% CI [1.92; 5.91], *p* < 0.001), reported more depression symptoms (*b* = 1.55, 95% CI [0.29; 2.82], *p* = 0.016) as well as less self‐control (*b* = −0.14, 95% CI [−0.27; −0.003], *p* = 0.045) and had more difficulties in emotion regulation (*b* = 6.07, 95% CI [2.16; 9.99], *p* = 0.002) than in the SCD class (Table 3). In the sensitivity analysis with the three‐class solution, there was no evidence for an effect of stress exposure on craving in the first two classes (class 1: *b* = 1.12, 95% CI [−3.12; 6.37], *p* = 0.674; class 2: *b* = 1.70, 95% CI [−3.95; 0.56], *p* = 0.140) and higher increase in craving in the stress than in the control group in the third class (*b* = 6.16, 95% CI [1.70; 10.63], *p* = 0.007). Since these results further suggested that the three‐class solution does not provide a better differentiation compared with the two‐class model, we did not further compare the three classes.

**TABLE 2:** Fit indices of finite mixture models with one to three classes.

Models	LL	AIC	BIC	Entropy
1 Class	−971.21	1954.43	1976.28	–
2 Classes	−955.08	1934.17	1977.87	0.301
3 Classes	−947.53	1931.06	1996.61	0.391

*Note*: *N* = 282.

Abbreviations: AIC, Akaike information criterion; BIC, Bayesian information criterion; LL, log likelihood.

**TABLE 3:** Latent class characteristics.

Latent class	Stress‐related craving decrease	Stress‐related craving increase	*b* [95% CI]	*p*
*N* (%)	137 (48.58)	145 (51.42)		

	*M* (SD)	*M* (SD)		
Craving (T0)	27.48 (11.89)	30.74 (11.89)		
Consumption (g)	19.32 (17.19)	20.07 (19.82)	0.75 [−3.71; 5.22]	0.740
Craving (T1)	26.75 (11.12)	35.32 (13.49)	5.37 [1.99; 8.75]	0.002
Anxiety	32.83 (7.46)	36.76 (9.40)	3.91 [1.92; 5.91]	0.001
Depression	5.23 (4.61)	6.79 (6.10)	1.55 [0.29; 2.82]	0.016
Self‐control	3.36 (0.60)	3.22 (0.58)	0.14 [−0.27; −0.003]	0.045
Drinking motives	10 (2.96)	10.71 (3.15)	0.71 [−0.01; 1.43]	0.052
Emotion dysregulation	67.71 (15.14)	73.78 (17.83)	6.07 [2.16; 9.99]	0.002

*Note*: Consumption: STLFB—Self‐Administered Timeline Follow Back; Anxiety: STAI‐T—State‐Trait Anxiety Inventory‐Trait anxiety; Depression: BDI—Becks Depression Inventory; Self‐control: BSCS—Brief Self‐Control Scale; Drinking Motives: DMQ—Drinking Motives Questionnaire; Emotion Regulation: DERS—difficulties in emotion regulation scale. b—regression coefficient; *p*—*p*‐value; g—Alcohol in grams; T1—Measurement timepoint + 1 min poststress exposure.

## DISCUSSION

The present study investigated the effect of stress exposure on alcohol craving in a nonclinical sample of young male individuals. The results revealed evidence for a stress‐induced increase in craving, which was associated with a decline in mood. Furthermore, there was evidence for a relationship between stress‐induced craving and biological stress reactivity (alpha‐amylase but not cortisol). Preliminary findings also suggested the existence of distinguishable subgroups in the effect of stress on craving.

The findings from this experimental study in a nonclinical sample are in line with observational and experimental studies that reported associations between stress exposure and alcohol craving in clinical samples for both males and females (Guinle & Sinha, 2020; Wemm et al., 2019). However, it should be noted that, in line with previous findings, the average effect of stress on craving was rather small. Unlike some previous studies which were primarily conducted in AUD samples (Seo & Sinha, 2014; Trautmann, Kräplin, et al., 2018a), we only found an association between biological stress reactivity and craving for alpha‐amylase but not for cortisol. Evidence from animal studies suggests that increases in alpha‐amylase, a marker of sympathetic activity caused by stress, are mediated by central noradrenergic signals (Ditzen et al., 2014). Furthermore, central arousal has been linked to the reinstatement of drug‐seeking behavior, which is also believed to be mediated by central noradrenergic actions (España et al., 2016). Lastly, blocking noradrenergic activity with adrenoceptor agonists in humans has been associated with a reduction in drug craving (Sinha et al., 2007). In light of these findings, it could be assumed that the association between alpha‐amylase and craving in our study is mediated by increased stress‐related arousal. A potential explanation for the lack of an association between cortisol and craving is that in clinical populations, repeated alcohol use can dysregulate the HPA axis resulting in reduced cortisol recovery and reactivity (Weckesser et al., 2025) and alter reward pathways, thereby strengthening the link between biological stress and craving (Koob & Le Moal, 2008; Sinha, 2008). By contrast, in nonclinical individuals, the HPA axis and reward pathways may remain intact, and craving may instead be influenced by psychological or situational factors. This is supported by evidence indicating that stress‐reactivity mechanisms differ between individuals at risk for addiction and those without clinical diagnoses (Lovallo, 2006). For example, Childs et al. (2011) found that nondependent drinkers show increased craving due to subjective stress perception rather than biological stress markers, a pattern we also observed in our study. Furthermore, the current results align with prior research indicating that while cortisol stress responses are governed by slower, hormonal regulatory mechanisms, psychological processes such as craving are more dynamic and influenced by a broader range of cognitive and emotional factors (Koob & Le Moal, 2008; Sinha, 2008). Sinha proposed that craving is closely tied to emotional states and may peak during acute stress but diminish as the individual adapts or reframes the stressor (Sinha, 2007). The observed transient increase in craving may reflect the psychological processing of the stressor. Initial stress exposure could heighten emotional distress, which intensifies craving as an immediate coping mechanism. However, as participants engage in cognitive or emotional regulation, craving may subside even if the underlying biological stress remains unresolved. This is supported by studies indicating that the subjective perception of stress often diminishes more quickly than the biological markers of stress (Heatherton & Wagner, 2011). While self‐reported craving represents an established and valid proxy of motivational processes underlying alcohol use (Sinha, 2008), it remains an indirect measure and does not always translate into actual drinking behavior. Although craving reliably predicts alcohol consumption in clinical samples (Serre et al., 2015; Thomas et al., 2011), studies involving nondependent participants often report weaker or inconsistent associations (Clay & Parker, 2018; Tartter & Ray, 2012), and in some cases, even inverse patterns among females (Bacon et al., 2015). Therefore, translating experimental findings on stress and craving to real‐world drinking behavior requires caution, but can be helpful to identify mechanisms that could be potential precursors of drinking initiation and serve as valuable targets of future studies. Our findings further highlight the complexity of stress‐craving relationships in nonclinical populations and emphasize the need for distinguishing different components of stress and its temporal dynamics.

In exploratory analyses, the present study suggested two distinct classes in the association between stress exposure and craving, with the first class showing an increase and the second class showing a decrease in craving following stress. Although, like described above, many studies describe associations between stress and alcohol‐related variables such as craving, the literature is rather mixed, with many studies finding no or negative associations with craving or alcohol use (Clay & Parker, 2018). Thus, the hypothesis of distinguishable subgroups and the relevance of moderators in the association between stress and alcohol craving appears very plausible. In our study, individuals belonging to the SCI were characterized by higher anxiety and depressive symptoms, higher levels of emotion dysregulation and lower self‐control compared with individuals for which craving decreased after stress exposure (SCD). What is also noteworthy is that both groups did not differ regarding average daily alcohol use. Thus, based on these findings, we hypothesize that in nonclinical samples, psychological variables related to maladaptive subjective processing of stress exposure might be at higher risk for stress‐related alcohol craving, independent of their drinking levels. Craving, in this context, may then serve as a mediating mechanism linking internal stressors to maladaptive coping behaviors. On the one hand, this is in line with the initially proposed theoretical model of stress being related to alcohol use tendencies at all levels of use, from initiation to excessive consumption (Lijffijt et al., 2014). On the other hand, this hypothesis is also supported by our finding that subjective (but not biological) stress reactivity was associated with an increase in alcohol craving, since the abovementioned psychological variables distinguish between latent classes of increased and decreased craving have all been related to higher subjective stress reactivity (Ilen et al., 2024). Craving likely serves as both a manifestation of psychological distress (e.g., anxiety and depression) and a driver of maladaptive coping mechanisms, such as alcohol use (Grüsser et al., 2006). Trait anxiety, as a stable characteristic, may predispose individuals to increase sensitivity to stressors, which, in turn, exacerbates craving as a mechanism for coping with emotional distress (Sinha, 2008). Similarly, depression marked by diminished reward processing and increased negative affect has been consistently linked to substance use (Grüsser et al., 2006) as a maladaptive attempt to self‐soothe (Koob & Le Moal, 2008). Low self‐control might be related to impaired executive functioning in the inability to suppress or resist experiences of cravings (Heatherton & Wagner, 2011). In summary, these variables are valuable targets for studying more personalized approaches in understanding the relationship between stress and alcohol‐related behaviors. It should be clearly noted that these findings must be interpreted with caution, not only because of their exploratory nature, but also because we did not find unequivocal evidence for two clear‐cut classes with great model fit. One reason might be the limited variance in study measures in this particular sample. However, it seems likely that there is heterogeneity in the effect of stress on craving and these findings might be of value for delineating novel hypotheses that can be tested in future studies.

While the present study provides valuable insights into the relationship between stress and alcohol craving, several limitations should be considered when interpreting the findings. First, the study focused exclusively on a nonclinical sample of young male individuals, which limits the generalizability of the results. This is particularly important in light of recent developments indicating a narrowing gender gap in alcohol use and AUD, with some subgroups of females experiencing, especially severe consequences of excessive consumption (White, 2020). Second, there was a large proportion of university students included in the study sample which might also has affected the current results as higher socioeconomic status (SES) is associated with more frequently alcohol drinking (Huckle et al., 2010). Third, the study was conducted in a controlled laboratory environment, which, although beneficial for internal validity, poses limitations for ecological validity. Laboratory‐induced stressors may not fully reflect the complexity and variability of real‐world stressors, and the mechanisms leading to craving in the laboratory may differ from those in everyday life. As a result, the influence of dynamic, contextual factors in stress‐craving interactions is likely underestimated. Fourth, the proportion of missing values for the biological measures of stress reactivity is somewhat larger than other variables as a result of data cleaning processes. Although this is common, the smaller sample size and a higher risk for selection bias have to be considered for the respective analyses. Finally, the anticipation of available alcoholic drinks might have influenced the absolute values of craving, which has to be considered for the correlational analyses within the stress group.

Given these limitations, future studies should aim to replicate the findings in more diverse samples, including nonmale participants, to explore potential gender differences in stress‐related alcohol craving. Additionally, research conducted in more naturalistic settings is essential to better capture the real‐world dynamics of stress and craving. Utilizing ecological momentary assessment (EMA) could allow researchers to measure these variables in real‐time and in participants' natural environments (Wray et al., 2014). This approach would facilitate a deeper understanding of the temporal and reciprocal influences between stress and craving, providing deeper insights into the processes involved. Ultimately, such efforts will enhance the development of targeted, evidence‐based interventions to prevent and treat maladaptive alcohol use behaviors.

Taken together, the present findings underscore the multifaceted nature of stress‐related alcohol craving in nonclinical populations and highlight the importance of integrating biological, psychological, and contextual perspectives to more accurately characterize underlying mechanisms and guide the refinement of future empirical investigations, methodological approaches, and translational prevention strategies.

## AUTHOR CONTRIBUTIONS

Sebastian Trautmann: Conceptualization; data curation; formal analysis; funding acquisition; investigation; methodology; project administration; resources; supervision; validation; writing—review and editing. Charlotte Wittgens: Data curation; formal analysis; investigation; project administration; writing—original draft. Markus Muehlhan: Conceptualization; methodology; writing—review and editing. Anja Kräplin: Methodology; supervision; validation; writing—review and editing. Fee Ona Fuchs: Investigation; writing—review and editing.

## FUNDING INFORMATION

The study was funded by the Deutsche Forschungsgemeinschaft (DFG): TR 1489/1‐1; project number: 383739866.

## CONFLICT OF INTEREST STATEMENT

The authors declare no conflicts of interest.

## Data Availability

The data that support the findings of this study are available from the corresponding author upon reasonable request.
